# Dr. Collier's Celsus

**Published:** 1832-01-01

**Authors:** 


### XIII.

### DR. COLLIER'S CELSUS. Text and Translation in two separate volumes, 1831.

IF the old Roman could rise from his tomb, if indeed he ever had one, and perceive the respect which is now paid to his memory, even among those barbarian tribes of Picts and Scots whom the masters of the world attempted to subdue and civilize, his spirit would be gratified! If the medical profession in this country has hitherto paid too little attention to the study of Celsus, they are now making up for their negligence—and if we can form an estimate of the demand by the supply, this elegant compilator is devoured most greedily by all ranks of the profession. The table at which we write is nearly covered with editions of Celsus, each endeavouring to aim at superior accuracy or cheapness. We have spoken of those sent forth by Dr. Milligan and Mr. Lee. Dr. Collier is certainly entitled to his share of public patronage on account of the learning and labour which he has expended on these two volumes, which are remarkably cheap, as well as neatly executed. It appears that the first edition was exhausted in sixteen months, when the author's interest as well as inclination prompted him to carefully revise and republish his favourite work, with such amendments as should render it more deserving of public approbation. He informs his readers that he has endeavoured to be "as literal as common sense would permit," requesting such of his readers as desire a closer translation to bear in mind that he "never undertook to murder Celsus, and then to subject him to minute dissection, in order that a few dead fibres of his mangled corse might be submitted to the inspection of 'cruel examiners;' but to transfer him to their notice physically and essentially, body and soul, with so much spirit infused into the translation as might at least give a faint idea of the living original." After some cutting remarks on translators, the worthy Doctor informs us that "sympathising with that class of medical students who need every encouragement, he has prepared an ordo verborum of the first and third books, after such a method, that 'he who runs may read,' which he shall direct the booksellers to supply separately at little more than the cost of print and paper."

In our last number we gave a specimen of Mr. Lee's translation. We shall here select a different place and give a specimen from each of the two authors, by which our readers may form some idea of their comparative merits.

"Ut alimenta sanis corporibus agricultura, sic sanitatem ægris medicina promittit. Hæc nusquam quidem non est. Siquidem etiam imperitissimæ gentes herbas, aliaque prompta in auxilium vulnerum, morborumque, noverunt. Veruntamen apud Græcos aliquanto magis quam in cæteris nationibus exculta est, ac ne apud hos quidem à prima origine, sed paucis ante nos seculis; utpote cum vetustissimus auctor Æsculapius celebretur. Qui quoniam adhuc rudem et vulgarem, hanc scientiam paulo subtilius excoluit, in Deorum numerum receptus est. Hujus deinde duo filii, Podalirius et Machaon, bello Trojano ducem Agamemnonem secuti, non mediocrem opem commilitionibus suis attulerunt. Quos tamen Homerus non in pestilentia, neque in variis generibus morborum aliquid attulisse auxilii, sed vulneribus tantummodo ferro et medicamentis mederi solitos esse, proposuit. Ex quo apparet, has partes medicinæ solas ab his esse tentatas, easque esse vetustissimas."

TRANSLATIONS.

LEE.

"As agriculture provides aliment to the sound body, so medicine does health to the sick. Indeed no part of the world is without this art. For the most uncultivated nations know the properties of herbs, and other prompt remedies for wounds and diseases. But it

COLLIER.

"As agriculture to those who are in health, holds out the expectation of aliment, so medicine promises to the sick a recovery from disease. There is not a spot on the habitable globe where the healing art has not some footing; for even the most uncivilized tribes have was cultivated by the Greeks, a little more than other nations, yet not even by them from the origin of that people, but a few ages before us; as it would appear Æsculapius is celebrated as their most ancient author, who, because he cultivated this art, hitherto rude and barbarous, a little more skilfully, was received into the number of their gods. Afterwards, his two sons, Podalirius and Machaon, having followed their general, Agamemnon, to the Trojan war, did not render little assistance to their fellow soldiers. But Homer has represented that they did not attempt to cure pestilence nor various other kinds of diseases, but were in the habit of dressing wounds by the knife and medicines only: by which it appears, they were accustomed to treat surgical cases only, and that this was the most ancient."

some knowledge of herbs, and other remedies easily procured for the relief of wounds and diseases. It has been advanced by cultivation, however, among the Greeks more than among other nations; nor with them from their first origin, but a few centuries only before our own time; for Æsculapius is celebrated as its most ancient author, and was deified for having more ingeniously cultivated a science, which, up to his time, had been devoid of arrangement and in low estimation. His two sons, Podalirius and Machaon, followed in the train of Agamemnon, the commander of the Trojan expedition, and afforded no inconsiderable assistance to their fellow soldiers in arms; not that Homer mentions them as curing the plague, or as treating any of the various kinds of disease; but describes them as in the habit of treating wounds only, by operations and medicine. So that it is manifest they practised these departments exclusively, and that they are the most ancient."

It will be abundantly evident that Dr. Collier has a great dislike to *literal* translation. Thus, in the first sentence, " promittit," in Celsus, is rendered into English by three words instead of one, viz. " holds out expectation." Promittit serves both for agricultura and medicina—and we see no reason why Dr. Collier might not have followed the example, and made one English verb (promise) serve for the two nominatives. Mr. Lee goes closer to the original, making the word " provide" answer for both purposes.* In the second sentence of Dr. Collier, we find the word " *nusquam*," in Celsus, translated thus: " not a spot on the habitable globe." In Celsus, " hæc nusquam quidem non est" contains twenty-one letters—Dr. C's. translation contains *sixty-nine* letters, or more than triple the quantity of type. It is clear that the Doctor is determined not to fall into the fault of *obscurity* by too much attention to *brevity*.

We have indulged in this little piece of verbal criticism for mere amusement, and do not by any means find fault with Dr. Collier's translation. Of the two versions, we would say that Dr. Collier's is more free and easy—Mr. Lee's more stiff and literal. But let us look, for one moment, to this celebrated opening sentence of the Roman sage, which has commanded ad-

* We would translate the sentence thus: " As agriculture promises food to the healthy, so medicine promises health to the sick." Here there are precisely the same number of letters in the original and in the translation, viz. 70, although the verb " promises" is obliged to be repeated in the English version, while it is understood in the Latin. The translation is strictly literal, and we leave it to our readers whether it is not as harmonious (it is certainly more terse and faithful) as the more wordy translation of Dr. Collier.

miration for nearly two thousand years. In our humble opinion, there is just as much analogy between agriculture and physic, as there is between a steamer and a stable. Agriculture multiplies the fruits of the earth; physic corrects the disorders of the body. Agriculture, in fact, bears a greater similitude to matrimony than medicine. The one reproduces vegetable life—the other animal.

Dr. Collier comes to the conclusion, that Celsus *must* have been a *practitioner* himself, else he could not have compiled so well from the writings of others. We have some doubts of the correctness of this inference. That Celsus *studied* medicine there can be no question; but that he should have actually *practised* it, there seems no positive proof afforded by the fact of an elegant and eloquent compilation. Suppose some very clever thesis of some very clever student were to turn up two thousand years hence—would it not be inferred that he was a *practitioner* of medicine, although he might never have had the care of a single patient? Our own opinion is, that Celsus was an accomplished scholar, a man who studied various sciences—and among others medicine—but who did not practise the healing art. He wrote well on military tactics and agriculture; ergo, by Dr. Collier's argument, he must have been a soldier at one time, and a farmer at another!! For our own parts, we think it would be much more easy for a literary man to compile a treatise on physic without being a physician, than to write on military tactics without ever mixing with cannon and gunpowder. That practice is not essentially necessary for elegant compilation, we could adduce some strong reasons—perhaps some striking examples. A clergyman (the Reverend Mr. Clark) wrote the best treatise on *naval* tactics that ever was penned, without ever having his foot on board of a ship. What will Dr. Collier say to this? The late Dr. J. M. Good compiled an eloquent treatise on medicine—the *worst parts* of which are those where he is swayed by his own very limited practice. The great bulk of his "*Study* of Medicine," as he, with much naiveté, styles it, was the result of *library* practice—and if it had all been derived from this source, it would have been better, perhaps, than it now is. But we shall let Dr. Collier give his own reasons for the conclusion to which he has come.

"If I may be allowed to record my opinion, I would venture to say, his own work and the habits of his order would go to prove he was not a *money-making Physician*, for he severely condemns wholesale dealers in disease, and declares it to be impossible for any one person to attend and do justice to a great number of patients: he was not a *Hospital Physician*, for he animadverts likewise upon that class of practitioners as *deficient in care and discernment*; neither was he a servile imitator of the practice of his contemporaries, for he authoritatively recommends several modes of treatment which he declares to be in his time universally neglected."—*Preface*, vi.

We have marked a few words in Italics, to shew that Dr. Collier is not very favourable to those physicians who have many patients—who make much money—or who are attached to hospitals. But our author ought to recollect that, generally speaking, physicians have *very few patients* for a long time before they come to have very many—that they are in the receipt of *very few fees* for many years before they accumulate a fortune—and that, although chance, interest, and nepotism, too frequently throw young men into the charge of hospitals, yet these schools are the very best for the acquirement of sound knowledge.

"What was he then? A literary charlatan, who compromised the interests of posterity, by authoritatively laying down precepts concerning the life and death of his fellow-creatures, without having repeatedly put those precepts to the test of experience? He could not do it. He would have been the laughing-stock of Rome. Let us examine the following passages: 'Ego autem medicamentorum dari potiones, et alvum duci non nisi rarò debere, concedo.' Lib. iii. cap. 6. What! Concession upon a practical point, emanating from a man who never practised? It must have been a modest concession with a vengeance! 'Ego tùm hoc puto tentandum, quùm parùm cibus,' &c. Lib. iii. cap. xi. If not a practitioner, of what consequence was it to the physicians of Rome, what he thought? 'Ego utique, si satis virium est, validiora; si parùm imbecilliora auxilia, præfero.' Lib. iii. cap. xxiv. On what could he have grounded his preference, if not on his own practical results in a number of cases? 'Ego experimentis quemque in se credere debere,' &c. Lib. iv. cap. xviii. 'Ego eundem quidem hominem posse omnia ista præstare concipio.' Lib. vii. Præf. If not a practitioner, he would not have formed such a conception. 'Ego autem cognovi, qui, succisâ linguâ,' &c. Lib. vii. cap xiii. sect. 4. 'Ego sic restitutum esse neminem memini.' Lib. vii. cap. vii. sect. 6. It were easy to subjoin fifty such passages; let these suffice.

Now could he write so at Rome, where it must be notorious whether he really practised or not? Or can it be conceived any man could write so exactly upon medical and surgical subjects without being versed in practice?"—*Preface*, vii.

There is nothing in any of these passages to prove that Celsus practised medicine. Nothing is more common than for a *student* of medicine to express strong opinions, probationary or condemnatory, of that which is done or said by the actual practitioner. Every inaugural dissertation exemplifies this. In the very next page but one to that whence we quoted the above passage, Dr. Collier affirms, that "the fixed purpose of his life (Celsus) seems to have been, *to gain knowledge and to transmit it to posterity.*" If this were the case, and it probably was so, what would Celsus care for the remarks of the Romans? One thing is clear, that the work is a *compilation,* and whether the author was a library or a general practitioner, it is of little use now to enquire.

Dr. Collier is sometimes obscure, though not from addiction to brevity. It would not be easy to decipher the Doctor's meaning in the following passage.

"He (Celsus) was no advocate for pharmaceutic medicine. Diet and the digestive organs were his watchwords. *Let our modern Celsus candidly confess his early obligations to the Roman*, and *save future historians the pain of inflicting censure: it would be a most righteous retribution, redounding to the honour of both.*"—*Preface.*

We believe that "glorious Johnny" was in the land of the living when Dr. Collier penned the above. If he alluded to Abernethy, (and we cannot imagine any other person to whom he can allude) we may safely say that that talented, but eccentric, practitioner was little indebted to Celsus, or to any other ancient writer, for his ideas or practices. We very much doubt whether he ever read a page of Celsus or Hippocrates in his life.

Cheap as is this edition of Celsus, Dr. Collier might have rendered it still cheaper, or, at all events saved himself some considerable expense, by the omission of plates, representing ancient baths, instruments, and characters, now totally useless. Of what possible utility to a medical book can be a large plan of the baths of Diocletian in Rome? baths never intended for medicinal purposes, but for the indulgence of luxury and debauchery among a degenerate and slavish people. It is true that the Roman Thermæ have been lauded by thoughtless travellers, on account of their magnitude and magnificence, while the pious Eustace has characterized the daily use of these baths among the Romans as a "*semi-virtue*," deserving of imitation in Britain!

"This SEMI-VIRTUE—this daily and promiscuous congregation of *both sexes* in Stygean hot-baths—this scene of indecency—this sink of sensuality, against which the edicts of Adrian and Aurelian were issued in vain—scenes which so scandalized (or rather mortified) the incestuous, murderous, meretricious AGRIPPINA, that she could not bear the idea of the Roman fair sex being on a par with herself in licentiousness—and, therefore, constructed FEMALE BATHS on the Viminal Hill, which, we may well believe, were little frequented :—Such are the *semi-virtuous* establishments which the simple, and, I have no doubt, pious EUSTACE bewailed the want of in his native land!"—*Change of Air, or Diary of a Philosopher, 2d Ed. p.* 158.

But this is a digression, and we shall now select a short chapter from Dr. Collier's volumes, as a sample of text and translation, from which we have no doubt the reader will come to the conclusion, that the author has executed his task with considerable ability.

CELSUS.

"QUEM INTERDIU VEL DOMESTICA VEL CIVILIA OFFICIA TENUERUNT, HUIC TEMPUS ALIQUOD SERVANDUM CURATIONI CORPORIS SUI EST. Prima autem ejus curatio exercitatio est, quæ semper antecedere cibum debet; in eo qui minùs laboravit et benè concoxit, amplior; in eo qui fatigatus est, et minùs concoxit, remissior. Commodè verò exercent, clara lectio, arma, pila, cursus, ambulatio; atque hæc non utique plana commodior est; siquidem meliùs ascensus quoque et descensus cum quâdam varietate corpus moveat, nisi tamen id perquàm imbecillum est: melior autem est sub divo quàm in porticu: melior, si caput patitur, in sole quàm in umbrâ: melior in umbrâ quam parietes aut viridaria efficiunt, quàm quæ tecto subest; melior recta quàm flexuosa. Exercitationis autem plerumque finis esse debet sudor, aut certè lassitudo quæ citra fatigationem, sit; idque ipsum modò minùs, modò magis faciendum est. Ac ne his quidem, athletarum exemplo, vel certa esse lex vel immodicus labor debet. Exercitationem rectè sequitur modò unctio, vel in

TRANSLATION.

"HE WHO IS DAILY OCCUPIED, WHETHER WITH PRIVATE OR PUBLIC AFFAIRS, OUGHT TO SET APART SOME PORTION OF HIS TIME FOR THE CARE OF HIS HEALTH. Now the chief means of preserving this is exercise, which ought always to precede a meal, more severe with him who has been studying less hard, and whose concoction is perfect; gentle with him who is exhausted, and who has concocted but in part. Reading aloud, martial weapons, the ball, running and walking, are means of exercise convenient enough; the last is more beneficial on ground not too level; for a slight ascent and descent affording more variety to the motion of the body, is preferable unless this be extremely weak. Exercise in the open air is better than that in a portico; better, if the head permit, in the sun than in the shade, better in the shade of walls and groves, than in that of a covered building; better in a straight than in a winding direction. Most generally it should be continued until some sweating ensues; or at least a lassitude not amounting to fatigue: sometimes to a greater, sole vel ad ignem; modò balneum, sed conclavi quàm maximè et alto et lucido et spatioso. Ex his verò neutrum, semper fieri oportet, sed sæpiùs alterutrum, pro corporis naturâ. Post hæc paulùm conquiescere opus est. Ubi ad cibum ventum est, nunquàm utilis est nimia satietas; sæpè inutilis nimia abstinentia: si qua intemperantia subest, tutior est in potione quàm in escâ. Cibus à salsamentis, oleribus, similibusque rebus meliùs incipit: tùm caro assumenda est, quæ assa optima aut elixa est. Condita omnia duabus de causis inutilia sunt, quoniam et plus propter dulcedinem assumitur, et quod modo par est, tamen ægriùs concoquitur. Secunda mensa bono stomacho nihil nocet: in imbecillo coascessit. Si quis itaque hoc parùm valet, palmulas pomaque et similia meliùs primo cibo assumit. Post multas potiones, quæ aliquantùm sitim excesserunt, nihil edendum est: post satietatem, nihil agendum. Ubi expletus est aliquis, faciliùs concoquit, si quicquid assumpsit, potione aquæ frigidæ includit: tùm paulisper invigilat, deindè benè dormit. Si quis interdiù se implevit, post cibum neque frigori neque æstui, neque labori, se debet committere: neque enim tam facilè hæc inani corpore quàm repleto nocent. Si quibus de causis futura inedia est, labor omnis vitandus est." 16.

and sometimes to a less extent. For them, there are not, as with wrestlers, any certain rules, nor ought their exercise to be immoderate. Exercise is rightly followed up sometimes by inunction, whether in the sun, or near the fire; sometimes by the bath, but in a very lofty, well lighted, and spacious apartment. But in truth, neither of these ought uniformly to be practised; but this or that more frequently, according to the nature of the constitution: afterwards a little rest is necessary. When meal-times arrive, surfeiting never does good; excessive abstinence often harm; but if intemperance be committed, it is safer in drink than in food. It is better to begin a repast with salsaments, vegetables, and other things of that nature: then, meat should be taken, which is best roasted or boiled. All ragouts are pernicious, for two reasons; because they are taken to excess on account of their agreeable flavour, and because even in moderation they are digested with difficulty. A dessert does no harm to a strong stomach, but turns sour in a weak one. He, therefore, whose health is indifferent, more properly takes his dates, orchard fruit, and the like, at an early period of the meal. After drinking considerably more than thirst requires, one should eat nothing; after a surfeit, one should do nothing. Whenever a person has eaten too heartily, he will concoct more easily by concluding his meal with a draught of cold water, remaining a short time awake, and then taking a good nap. He who has fed too heartily, ought neither directly afterwards to expose himself to cold, nor to heat, nor to labour; for these things are not so hurtful when digestion is suspended, as they are on a full stomach. Therefore, when, no matter from what cause, it becomes expedient that we should fast, all labour should be avoided." 17.

We have a high respect for Dr. Collier's classical acumen and literary research, and though we have thrown out a few trifling critical remarks, we strongly recommend the work to our professional brethren.

